# Usefulness of cerebrospinal fluid analysis in dogs and cats with suspected intracranial disease and normal magnetic resonance imaging

**DOI:** 10.3389/fvets.2025.1583988

**Published:** 2025-06-20

**Authors:** Susana R. Monforte Monteiro, Luisa De Risio, Lisa Alves, An E. Vanhaesebrouck

**Affiliations:** ^1^Department of Veterinary Medicine, University of Cambridge, Cambridge, United Kingdom; ^2^Linnaeus Group Clinical Research and Excellence, Linnaeus Veterinary Limited, Shirley, United Kingdom

**Keywords:** brain, diagnostic, imaging, clinical pathology, inflammation

## Abstract

Cerebrospinal fluid (CSF) analysis is a common diagnostic tool in the investigation of neurological presentations. Whether its routine use after every brain magnetic resonance imaging (MRI) is warranted is debated amongst clinicians, and its usefulness after a normal MRI has not yet been examined. To investigate whether CSF analysis affected the final diagnosis in dogs and cats with suspected intracranial disease in the presence of unremarkable magnetic resonance imaging (MRI), clinical, imaging and laboratory records of dogs and cats with suspected intracranial disease, unremarkable MRI and CSF analysis were reviewed in this multi-center retrospective study. Of 593 animals, (533 dogs and 60 cats), 17 dogs (3%) had abnormal CSF, nine of these demonstrating pleocytosis (with or without elevated microprotein) and eight showing hyperproteinorrachia alone. In only five of these dogs (0.8% of the total cohort) was the final diagnosis and/or treatment meaningfully affected by CSF findings: three diagnosed with inflammatory brain conditions and two had undetermined diagnoses, with corticosteroids initiated following abnormal CSF results. No cats in this population had an abnormal CSF. All dogs with a diagnosis based on abnormal CSF results had an abnormal neurological examination. In this population, CSF analysis was unlikely to reveal an undiagnosed intracranial condition following an unremarkable brain MRI, particularly in dogs presenting with a normal neurological examination. In dogs presenting with an abnormal neurological examination or a high suspicion of inflammatory disease, CSF evaluation following normal MRI is more likely to be diagnostically valuable.

## 1 Introduction

Cerebrospinal fluid (CSF) analysis is a commonly used diagnostic tool in veterinary neurology. Routine evaluation includes cytological assessment and biochemical characteristics. An increase in the total nucleated cell count (TNCC) in the CSF is considered consistent with neuroinflammation or infection (1). In certain tumor types, neoplastic cells can sometimes also be found (2). An increase in CSF microprotein, whether accompanied or not by pleocytosis, is a relatively common finding in intracranial central nervous system (CNS) disease, but is considered to be non-specific (3, 4). The lack of CSF abnormalities in dogs with recurrent epileptic seizures can also be supportive of a diagnosis of idiopathic epilepsy (5), although the diagnostic value of routine CSF analysis in these cases has been questioned (6, 7).

CSF evaluation can be paired with MRI in the investigation of possible intracranial CNS conditions. Not uncommonly, MRI is normal in patients with clinical disease. This can potentially be due to the presence of lesions that are too small to be detected, mild and diffuse INFILTRATION, or limited disturbances of the blood brain barrier so that the lesion cannot be detected even with the use of gadolinium-based contrast. In veterinary medicine, it is currently unknown whether CSF is expected to aid in reaching a final diagnosis in these cases, and this is pertinent information when balancing benefits and risks. Although complications of CSF retrieval are very rare, they can occur and be serious or fatal (8–11). In humans, guidelines for CSF retrieval and analysis have been described (12, 13), but no guidelines have been defined in veterinary medicine, and the decision to sample CSF is largely clinician dependent, with some taking CSF routinely and others in only selected cases. The aim of this study was to investigate whether routine CSF evaluation affected the final diagnosis and/or treatment in dogs and cats with intracranial disease in the presence of a normal MRI.

## 2 Materials and methods

### 2.1 Dogs and cats

Medical records of dogs and cats presenting between 2015 and 2019 with clinical signs and/or history compatible with an intracranial neuroanatomical localization were retrieved from two veterinary referral centers (Queen's Veterinary School Hospital, University of Cambridge, UK and Animal Health Trust, Newmarket, UK).

Dogs and cats were included if they fulfilled the following inclusion criteria: (1) clinical signs and/or history compatible with intracranial neurological disease, (2) full detailed neurological examination by a board-certified neurologist or residency-trained neurologist, (3) normal MRI study of the head including T1-weighted pre- and post-contrast sequences (brain, bullae, and cranial nerves assessed), reviewed by a board-certified radiologist and neurologist, and (4) CSF analysis from cerebellomedullary tap (TNCC, cytology, and microprotein).

### 2.2 Retrieved data

Collected data included species, breed, age at diagnosis, sex, reason for presentation, and presence or absence of signs on neurological examination. Information regarding final diagnosis, treatment, and outcome was retrieved from clinical records.

MRI and CSF tap were performed under general anesthesia. MRI of the head was acquired with either a low-field magnet (0.2 Tesla) or a high-field magnet (1.5 Tesla). MRI studies included a minimum of T1-weighted (pre- and post-contrast) and T2-weighted sequences in transverse and at least either sagittal or dorsal planes, and a transverse fluid-attenuation inversion recovery. Gradient echo was also included in most studies. A study was considered normal if it did not show a structural abnormality, mass lesion, abnormal signal intensity, or a combination of these that could account for the patient's initial presentation in pre- or post-contrast sequences (14). Ventricular asymmetry and/or lack of septum pellucidum were considered as “normal” findings in brachycephalic dogs.

CSF was obtained from the cerebellomedullary cistern and considered normal if TNCC was <5/μl and if microprotein was <0.30 g/L (1). No corrections for blood contamination were performed, in accordance with the most recent guidelines (15).

### 2.3 Statistical analysis

A Fishers exact test and risk ratio were performed to evaluate the influence of the neurological examination in the decision of a final diagnosis and/or clinical management. A value of *p* < 0.05 was considered statistically significant.

## 3 Results

Five-hundred and ninety-three animals met the inclusion criteria comprising 533 dogs and 60 cats. Baseline characteristics can be found in Supplementary Table S1. The most common presentations for animals with a normal MRI were epileptic seizures, representing 60% of the animals (352/593), followed by vestibular signs representing 13% (76/533). A list of other presentations is detailed in Supplementary Table S1. Thirty-one percent of dogs (164/533) and 48% of cats (29/60) had an abnormal neurological examination at presentation (Tables 1, 2). Only 35 cases had a low-field MRI (32 dogs and three cats), with all the remaining having their study performed in the high-field scanner.

**Table 1:** Cases in which the abnormal CSF did not change the diagnosis and management.

**Cases**	**Neurological examination**	**CSF analysis**	**Diagnosis**
1	Abnormal	Hyperproteinorrachia (0.45 g/L) TNCC 1/μl RBC 16/μl	Idiopathic cranial polyneuropathy
2	Abnormal	Hyperproteinorrachia (0.39 g/L) TNCC 2/μl RBC 1/μl	Idiopathic epilepsy
3	Abnormal	Hyperproteinorrachia (0.41 g/L) TNCC 2/μl RBC 11/μl	Open (paroxysmal episode/seizure)
4	Abnormal	Hyperproteinorrachia (0.58 g/L) TNCC 3/μl RBC 5,600/μl	Idiopathic epilepsy
5	Abnormal	Hyperproteinorrachia (0.42 g/L) TNCC 1/μl RBC 0/μl	Idiopathic epilepsy
6	Abnormal	Hyperproteinorrachia (0.62 g/L) TNCC 4/μl RBC 1/μl	Idiopathic cranial polyneuropathy
7	Abnormal	Mixed pleocytosis (TNCC 6/μl) Total protein 0.16 g/L RBC 2/μl	Idiopathic epilepsy
8	Abnormal	Hyperproteinorrachia 0.63 g/L TNCC 3/μl RBC 0/μl	Open (paroxysmal episode/seizure)
9	Abnormal	Mononuclear pleocytosis (TNCC 9/μl) Total protein 0.28 g/L RBC 916/μl	Idiopathic vestibular
10	Normal	Mixed pleocytosis (TNCC 19/μl) Hyperproteinorrachia (0.58 g/L) RBC 4,523/μl	Idiopathic vestibular
11	Normal	Neutrophilic pleocytosis (TNCC 12/μl) Hyperproteinorrachia (0.87 g/L) RBC 1,079/μl	Idiopathic epilepsy
12	Normal	Mononuclear pleocytosis (TNCC 14/μl) Unable to access protein RBC 6,800/μl	Idiopathic epilepsy

**Table 2:** Cases in which the abnormal CSF changed the diagnosis and management.

**Cases**	**Neurological examination**	**CSF analysis**	**Diagnosis**
1	Abnormal	Mononuclear pleocytosis (TNCC 46/μl) TP 0.31 g/L RBC 3/μl	Idiopathic generalized tremor syndrome
2	Abnormal	Hyperproteinorrachia (1.2 g/L) TNCC 3/μl RBC 3/μl	Open (dog with abnormal mental status and multiple cranial nerve deficits)
3	Abnormal	Mononuclear pleocytosis (TNCC 47/μl) Total protein 0.27 g/L RBC 9/μl	Meningoencephalitis of unknown origin
4	Abnormal	Mononuclear pleocytosis (TNCC 14/μl) Total protein 0.16 g/L RBC 11/μl	Idiopathic generalized tremor syndrome
5	Normal	Mononuclear pleocytosis (TNCC 15/μl) Total protein 0.23 g/L RBC 1/μl	Open (dog with history of suspected vestibular signs)

Five-hundred and seventy-six animals had a normal MRI and CSF, and 17 animals (2.9% of all animals; equivalent to 3.2% of dogs) had normal MRI and abnormal CSF. One dog (3%) that was investigated with a low-field MRI presented abnormal CSF (1/32), and 16 dogs (3%) that had a high-field MRI presented an abnormal CSF (16/501). None of the dogs and cats were reported to suffer from a deterioration of neurological status post-CSF retrieval.

All 17 cases in which an abnormal CSF following normal MRI was found were dogs; no cats were found to have abnormal CSF following normal MRI. In these 17 dogs, abnormalities found in routine evaluation of the CSF included elevated microprotein (*n* = 10; range 0.39–1.2 g/L) and pleocytosis (*n* = 9; TNCC 6–47/μl). When CSF analysis was abnormal, it did not alter the final diagnosis or treatment in 12 of the 17 cases (71%) but did so in five of the cases (29%). The 12 cases in which the final diagnosis was not altered, were diagnosed with idiopathic epilepsy (*n* = 6), idiopathic vestibular syndrome (*n* = 2), idiopathic cranial polyneuropathy (*n* = 2), and undetermined single paroxysmal events (*n* = 2; further details–see Table 1). CSF abnormalities in these cases were considered relatively mild (TNCC 6–19/μl; microprotein 0.39–0.87 g/L) and in five of the cases there was blood contamination, ranging from 916 to 6,800/μl (Table 1). In dogs diagnosed with epileptic seizures, three of them were reported to experience seizure activity [single seizure (*n* = 1), cluster seizures (*n* = 1), or status epilepticus (*n* = 1)] in the 48 h prior to CSF retrieval. Nine of the 12 cases presented with abnormalities on neurological examination, including tremors, vestibular signs, cranial nerve deficits, or mild generalized proprioceptive ataxia (for more details see Supplementary materials).

Within the animals with abnormal CSF, in only five of these (0.84% of total animals; 0.94% of total dogs), was the final diagnosis and/or treatment altered based on those results. In these cases, routine CSF analysis revealed either mild-to-moderate mononuclear pleocytosis (*n* = 4, 14–47/μl) or marked hyperproteinorrachia (*n* = 1, 1.2 g/L; Table 2). These patients were diagnosed with idiopathic generalized tremor syndrome (*n* = 2), meningoencephalitis of unknown origin (MUO; *n* = 1) and in two cases the diagnosis remained undetermined (*n* = 2; Table 2). Where a diagnosis was made, animals were treated accordingly. In the two cases in which the diagnosis remained undetermined, the clinician elected to institute corticosteroid treatment based on CSF results. Determination of efficacy of the corticosteroid treatment in these two cases was not possible, as one case did not show outward signs at presentation and the other was lost to follow-up.

A secondary aim of the study was to assess whether the presence of abnormalities on the neurological examination would influence the likelihood that CSF would alter the subsequent clinical management of the dog. In the group of animals for which abnormal CSF results altered the diagnosis and/or treatment, all cases except for one had an abnormal neurological examination (Table 2). Nine of 12 cases in which abnormal CSF did not alter the diagnosis and/or treatment presented neurologically abnormal (Table 1). Of these, six had a history of seizures/paroxysmal event, and presented with signs such as reduced menace response and proprioceptive ataxia, which were presumed to be post-ictal in origin. Two other dogs were diagnosed with idiopathic cranial polyneuropathy, and one with idiopathic vestibular syndrome.

Evaluation of the data shows that CSF analysis meaningfully altered the final diagnosis and/or clinical management in 2% (4/193) of patients with abnormal neurologic examination compared to 0.25% (1/400) with a normal neurological examination. When assessing dogs only, CSF analysis changed the subsequent clinical management in 2.4% (4/164) vs. 0.27% (1/369) of dogs with abnormal and normal neurologic examination, respectively—risk ratio = 9 (CI 95% 1.0137–79.9016; Fisher exact *p* = 0.0331). No cats with a normal MRI had an abnormal CSF analysis, irrelevant of their neurological examination.

## 4 Discussion

In dogs and cats with a neurological presentation suggestive of an intracranial neuroanatomical localization combined with a normal complete MRI study, the likelihood of routine CSF analysis (cytological evaluation and protein analysis) revealing an undiagnosed intracranial condition is low. The results of this study show that routine CSF analysis after every normal MRI in a dog or cat with a normal neurological examination is questionable, but that in the presence of a normal MRI study, with an abnormal neurological examination or a high suspicion of an inflammatory disease, routine CSF analysis is more likely to lead to a diagnosis and consequential change in management.

In only 5 out of 593 cases was the choice of treatment meaningfully altered following abnormal CSF results, and in only three of those cases a definitive final diagnosis could be determined following abnormal CSF. Of note, these three cases all had an abnormal neurological examination at presentation. Animals with an abnormal neurological examination were nine times more likely to have a CSF result changing the subsequent clinical management. This was statistically significant, but the confidence interval was fairly wide. The rarity of finding abnormal CSF in an animal with normal neurological examination and normal MRI questions the utility of CSF sampling every such case.

This study found CSF evaluation following normal MRI to be most useful in the diagnosis of inflammatory conditions, e.g., cases with MUO and idiopathic tremor syndrome. This is in agreement with a recent study looking into the role of routine CSF analysis in the diagnosis of vestibular disease found no obvious benefit in performing the test, except for dogs with inflammatory conditions, such as MUO (16). Meningoencephalomyelitis of unknown origin in dogs and cats can present with a normal MRI and the diagnosis can be based on an abnormal CSF alone (~20% of canine cases in literature) (17–20). Most dogs and cats with MUO appear to present with an abnormal neurological examination, with deficits depending on lesion(s) localization. Two dogs in this study were diagnosed with idiopathic generalized tremor syndrome. Compatible with the findings in the cases in this study, the veterinary literature reports that MRI studies are often unremarkable, and CSF abnormalities is a common finding in these cases (21).

In this study, no cats with a normal MRI had an abnormal CSF analysis, with or without an abnormal neurological examination. In another study, only 4% of 70 cats with epileptic seizures and a normal MRI were reported to show CSF abnormalities (22). It is possible that if our feline population was equivalent in numbers to our canine population, CSF abnormalities, whether leading to or not leading to alteration of the final diagnosis and/or treatment, might be found.

In our study, 12 dogs had an abnormal CSF analysis that did not result in an alteration in the final diagnosis and/or treatment. Half (*n* = 6) of these patients presented with a history of seizures. Routine CSF analysis has been previously recommended in the investigations for the seizuring patient: the Epilepsy Task Force considers an unremarkable CSF analysis as part of Tier II confidence level for the diagnosis of idiopathic epilepsy in dogs (5). However, seizure activity can itself lead to CSF abnormalities (often mild) on routine analysis (11%−15% prevalence) (23), and this does not appear to impact the final diagnosis (3, 6, 7). Similarly, in this study, an abnormal CSF did not change the diagnosis of idiopathic epilepsy in any of the dogs with findings consistent with this condition, whether or not they had an abnormal neurological examination (details–Table 1). In two dogs with abnormal CSF, an increase of total protein on CSF might have strengthened the diagnosis of idiopathic cranial polyneuropathy. Two other dogs were, despite changes in the routine CSF analysis, still diagnosed with idiopathic vestibular syndrome. CSF changes in these dogs were considered relatively mild and these animals followed the typical presentation of the suspected condition, which might have contributed to the decision of the clinician to disregard the abnormal CSF result. For two dogs with an open diagnosis following single paroxysmal events, mild hyperproteinorrachia did not alter the final diagnosis or treatment, and none of the dogs re-presented to the referral hospital. It is of note that in four of the 12 dogs with abnormal CSF in which the clinical management was not meaningfully altered, mild to severe blood contamination was present in the CSF and although the samples were still considered diagnostic and with no requirement for corrections (as below the reported 13,200 cells/μl) (15), this might have contributed to the clinical decision of overlooking the mild changes found. Thus, based on the results of this study, mild changes in routine CSF analysis do not necessarily alter the final diagnosis or treatment plan in dogs with either a normal or abnormal neurological examination. Most commonly, this appears to be the case when the CSF abnormalities are mild and are in association with a typical clinical presentation that corresponds to a characteristic condition, or if the event was a single event.

In people, clear guidelines exist for the performance of CSF retrieval via lumbar puncture, the most common method to obtain CSF in humans (12, 13). The main indication for the test is suspicion of CNS infection or inflammation, such as bacterial meningitis or viral meningoencephalitis, autoimmune encephalitis, but also subarachnoid hemorrhage, CNS lymphoma, and leptomeningeal metastatic disease (12). Less commonly, it is also performed to support the diagnosis of Guillain-Barre syndrome, multiple sclerosis or vasculitis (12, 13).

In dogs and cats, as well as in people, downstream tests of CSF, often performed following abnormal routine CSF, can be useful for bacterial and fungal cultures (1), and for CSF qPCR for infectious diseases following the detection of an inflammatory pattern on CSF (1, 12). Equally, if cytology detects malignant cells, flow cytometry and immunophenotyping can add to the characterization of the neoplastic process (1). Occasionally routine CSF can be considered to be unremarkable but can still be clinically useful. For example, in dogs with infectious meningoencephalomyelitis, about 19% can have a normal CSF, but still have positive PCR results for infectious agents (20).

More recently, the use of CSF biomarkers to aid the diagnosis and prognosis of neurological conditions has been recognized in both people and animals. Several potential markers, including specific proteins and metabolites, such as lactate or glial fibrillary acidic protein have been described in dogs (24–34), but are not used routinely in a clinical setting. In humans, some CSF biomarkers are now used as diagnostic tests for specific diseases (8). In future, these biomarkers will likely also become available to veterinarians in the clinic. The clinical availability of these biomarkers might lead to an increase in the number of cases in which a clearer clinical justification for CSF collection exists.

Limitations of this study include the use of different MRI machines (both low and high field) as it was a retrospective study, the possibility of subtle lesions being missed in the low field MRI. However, the same percentage of dogs with an abnormal CSF and normal MRI was seen in both low and high-field studies (3%). Another limitation was the lack of final histological diagnosis to confirm the clinical suspicions. The decision to take CSF was clinician dependent, with some clinicians always taking CSF (in the absence of contraindications) and others only taking CSF if they have a high suspicion that CSF will be useful. The alteration of the treatment plan following CSF results was also clinician dependent and could have been different had another clinician been managing the case. Lastly, our cat population was of only 60 cats, making it more difficult to draw conclusions in cats alone considering the smaller sample size when compared to the much bigger canine population in our study. Further studies, with a bigger feline population are needed to confirm the trend found.

## 5 Conclusion

In conclusion, it is unlikely that routine CSF analysis will lead to an altered final diagnosis or treatment in dogs and cats with an unremarkable MRI, particularly if they present with a normal neurological examination. Therefore, its routine use in these cases is questionable, and might instead need to be based on clinical suspicion. However, in animals with an abnormal neurological examination consistent with intracranial disease, or in cases with a high suspicion of an inflammatory brain disease, routine CSF evaluation following normal MRI could be of more value.

## Data Availability

The raw data supporting the conclusions of this article will be made available by the authors, without undue reservation.
