# Supplementary material for: Usefulness of cerebrospinal fluid analysis in dogs and cats with suspected intracranial disease and normal magnetic resonance imaging
Source: Front Vet Sci. 2025 Jun 20;12:1583988. doi: 10.3389/fvets.2025.1583988 (PMC12226868; doi:10.3389/fvets.2025.1583988)
Supplement: Supplementary file 1 [file Table_1.docx]

Supplementary Material

# Supplementary Data

| **Characteristics** | | **Number/type** |
| --- | --- | --- |
| **Species** | Dog | 533 |
|  | Cat | 60 |
| **Breed** | Over 50 | Labrador |
|  | 25-50 | Border collie, Crossbreed, Cocker spaniel, DSH |
|  | 10-25 | JRT, Border Terrier, ESS, Pug, Beagle, GSD, Chihuahua |
|  | <10 | 74 other breeds of dogs, and 7 breeds of cats |
| **Gender** | Male | 361: 331 dogs + 30 cats |
|  | Female | 232: 202 dogs + 30 cats |
| **Neutering status** | Neutered | 273 (male dogs), 30 (male cats), 146 (female dogs), 27 (female cats) |
|  | Entire | 81 (male dogs), 56 (female dogs), 3 (female cats) |
| **Size (dogs)** | <15kg | 149 |
|  | 15-30kg | 232 |
|  | >30kg | 152 |
| **Age** | Average (range) | Dog = 5yr (3m-13.5yr); Cat = 5yr (9m-16yr) |
| **Complaint** | Seizures | 352 (326 dogs + 26 cats) |
|  | Vestibular | 76 (57 dogs + 19 cats) |
|  | Generalized Tremors (persistent or episodic) | 16 (13 dogs + 3 cats) |
|  | Paroxysmal episodes | 39 (37 dogs + 2 cats) |
|  | Abnormal behavior | 34 (33 dogs + 1 cat) |
|  | Head tremor | 25 (24 dogs + 1 cat) |
|  | Fly catching/ star gazing | 9 (all dogs) |
|  | Cranial nerve abnormalities | 22 (19 dogs + 3 cats) |
|  | Lethargy | 3 (all dogs) |
|  | Abnormal sensation (hyperesthesia, facial rubbing) | 1 (cat) |
|  | Multifocal | 7 (4 dogs + 3 cats) |
|  | Blindness | 7 (6 dogs + 1 cat) |
|  | Ataxia | 2 (all dogs) |

**Supplementary Table 1** – Baseline characteristics of the population and initial complaint. DSH (Domestic Short Hair), JRT (Jack Russel Terrier), ESS (English Springer Spaniel), GSD (German Sheppard Dog)
